# Change in the intestinal bacterial community structure associated with environmental microorganisms during the growth of *Eriocheir sinensis*

**DOI:** 10.1002/mbo3.727

**Published:** 2018-10-11

**Authors:** Chenhe Wang, Yanfeng Zhou, Dawei Lv, You Ge, Huan Li, Yang You

**Affiliations:** ^1^ Wuxi Fishery College Nanjing Agricultural University Wuxi China; ^2^ Scientific Observing and Experimental Station of Fishery Resources and Environment in the Lower Reaches of the Changjiang River Ministry of Agriculture Freshwater Fisheries Research Center CAFS WuXi China; ^3^ Nextomics Biosciences Co., Ltd Wuhan China

**Keywords:** environmental microorganisms, *Eriocheir sinensis*, intestinal microbes, network analysis

## Abstract

As an important organ to maintain the host's homeostasis, intestinal microbes play an important role in development of the organism. In contrast to those of terrestrial animals, the intestinal microbes of aquatic organisms are affected by environmental microorganisms (including water microorganisms and sediment microorganisms). In the present study, the compositional differences of intestinal microbes in three representative developmental stages of the Chinese mitten crab (*Eriocheir sinensis*) were studied. Meanwhile, network association analysis, and visualization of the water microorganisms of the crabs’ habitat, the environment microorganisms in the pond, and the intestinal microbes, was carried out. The results showed that the gut microbiota diversity index decreased continuously with age, and the four bacteria of *Aeromonas* (Proteobacteria), Defluviitaleaceae (Firmicutes), *Candidatus Bacilloplasma* (Tenericutes), and *Dysgonomonas* (Bacteroidetes) were the “indigenous” flora of the crab. In the network‐related analysis with the environment, we found that as the culture time increased, the effect of environmental microorganisms on the intestinal microbes of crabs gradually decreased, and the four “indigenous” bacteria were always unaffected by the environmental microorganisms. The results of this study identified the core bacteria of the crab and, for the first time, studied the relationship between intestinal environmental microorganisms, which will aid the practical production of crabs and will promote research into the relationship between specific bacteria and the physiological metabolism of crabs.

## 1 INTRODUCTION

As one of the oldest and most economically valuable species, the Chinese mitten crab (*Eriocheir sinensis*) is cultured in the provinces of Jiangsu, Hubei, and Anhui and occupies an important position in the aquaculture industry in China. With the success of artificial seeding in the 1990s, the crab industry developed rapidly. To pursue high economic efficiency, crabs were farmed at high‐density, which led to the frequent occurrence of various diseases. With the growing awareness of ecology and the pursuit of the ecological value of commodities, eco‐aquaculture has become an inevitable trend in crab farming.

Intestinal microorganisms, which play an important role in the host's entire physiological process, have undergone long‐term development with the host (Bates, Mittge, Kuhlman, & Baden, 2006). Gut microbes play an important role in helping the host to maintain normal physiological processes, assisting nutrient absorption, and assisting in host immunity (Bäckhed, Ding, Wang, & Hooper, 2004; Bäckhed, Manchester, Semenkovich, & Gordon, 2007; Cebra, 1999; Sonnenburg, Xu, Leip, & Chen, 2005). Therefore, host and gut microbes maintain a homeostasis during long‐term evolution, and a stable gut microbial community is crucial to the host. However, there are many factors that affect gut microbes, such as host genotype, different stages of host development, different physiological states, life habitats, and eating habits (Bakke, Coward, Andersen, & Vadstein, 2015; Leamaster, Walsh, Brock, & Fujioka, 2010; Miyake, Ngugi, & Stingl, 2015; Mouchet, Bouvier, Bouvier, & Troussellier, 2012; Rungrassamee, Klanchui, Chaiyapechara, & Maibunkaew, 2013; Yan, Gast, & Yu, 2012). Studying the factors and rules influencing the gut microbes is significant to help coordinate research into the host and gut microbe interaction (Chaiyapechara, Rungrassamee, Suriyachay, & Kuncharin, 2012; Li, Zheng, Tian, & Yuan, 2007; Liu, Wang, Liu, & Wang, 2011; Yan et al., 2012). The study of gut microbes in aquatic animals has focused on fish; however, little information is available for crustaceans, especially for the Chinese mitten crab. To date, studies on gut microbes in crabs have mainly focused on the core bacteria and the relationship between gut microbes and diseases (Chen, Di, Wang, & Li, 2015; Li et al., 2007; Shen, Zang, Song, & Ma, 2017). Only one study has examined the relationship between gut microbes and the water environment in different organs of crab (Zhang, Sun, Chen, & Cai, 2016). How intestinal microorganisms in crabs are regulated during the breeding cycle is an unanswered question.

The impact of aquatic ecosystems on aquatic animals is enormous. Chinese mitten crabs mainly live in the bottom water in the breeding pond; therefore, the influence of the sediment cannot be neglected. Previous studies focused on the composition of microbes in the intestine of the crabs and its relationship with the aquatic environment (Chen et al., 2015; Zhang et al., 2016). However, the associations between sediment microorganisms, water environment microorganisms, and intestinal microbes in crabs are unknown.

Based on previous studies, we summarized the existing research on gut microbes of the crab and drew lessons from the study of intestinal microorganisms in fish and other crustaceans. The gut microbes of crabs were determined at three time points during the breeding cycle using 16s rRNA high‐throughput sequencing, together with correlation analysis of environmental microorganisms in the breeding ponds. The results were used to study the process of gut microbe assembly and to explore the shared and differential bacteria between gut microbes and environmental microorganisms during the breeding cycle. Our results provide reliable data support for subsequent studies on microbial community functions and ecological aquaculture techniques.

## 2 MATERIALS AND METHODS

### 2.1 Sample collection and illumina high‐throughput sequencing of bacterial 16S rRNA genes

From May to September 2017, crab samples were collected from ponds near Yangcheng Lake at three times points (Table 1). The groups of crabs had no significant differences in their specifications. The collected Chinese mitten crabs (*E. sinensis*) were transported to the laboratory as quickly as possible. After measuring the crab index, the surface of the crab was washed with sterilized water and 75% alcohol and then left for 3–5 min at room temperature. The entire intestine of the crabs was dissected out and placed in sterile tubes. The DNA was isolated from the intestines using an EZNA Tissue DNA kit (OMEGA Bio‐Tek, Norcross, GA, USA). The total DNA obtained from three identical crabs (no significant differences in individual size) was combined. The handling of the Chinese mitten crabs strictly followed the recommendations in the standard of care and use of experimental animals. The entire experimental procedure was approved by, and was under the supervision of, the Freshwater Fisheries Research Center, Chinese Academy of Fishery Sciences (FFRC, CAFS).

**Table 1:** Sample information for research

Time of sample collection	Sample type	Age	Weight of crab (g)	Sample group number	Number of samples^a^	BioProject number (Genbank)
May‐2017	Female crab	Juvenile	24.11 ± 6.93	YF1	9	PRJNA451256
	Male crab	Juvenile	30.48 ± 6.12	YM1	9	
	Water	–	–	YS1	3	PRJNA451312
	Sediment	–	–	YT1	3	
July‐2017	Female crab	Young	59.69 ± 9.29	YF2	9	PRJNA451256
	Male crab	Young	74.99 ± 8.90	YM2	9	
	Water	–	–	YS2	3	PRJNA451312
	Sediment	–	–	YT2	3	
September‐2017	Female crab	Adult	87.73 ± 8.59	YF3	9	PRJNA451256
	Male crab	Adult	110.91 ± 13.80	YM3	9	
	Water	–	–	YS3	3	PRJNA451312
	Sediment	–	–	YT3	3	
April‐2017	Origin water	–	–	QS1	2	PRJNA451312

*Note*

a Nine crab samples were divided into three groups. Each group was extracted for total DNA and mixed in equal amounts.

Water samples were collected at three sampling sites using a sterile container to take a total of 1 L from the pond for the extraction of water environment microorganisms. Crabs live in Yahu Lake, Qinghai province, before entering pond farming; therefore, water was collected from Yahu Lake as the water environment for the crab. Large suspended solids were removed using 5‐μm qualitative filter paper, and then the filtrate was vacuum‐filtered through a 0.22‐μm polycarbonate membrane (Millipore, Billerica, MA, USA). The membrane was used for bacterial genomic DNA purification by using a Water DNA Kit D5525 (OMEGA Bio‐Tek).

Sediment samples from the pond were collected using a Peterson grasp device with an open area of 1/40 m^2^. The collected sediment samples were stored in a sterile tube. The bacterial genomic DNA was collected using a Mag‐Bind^®^ Soil DNA Kit M5635 (OMEGA Bio‐Tek).

The V4 region of 16S rRNA gene was amplified using polymerase chain reaction (PCR) from all the bacterial genomic DNA that was harvested from fecal samples using barcoded fusion primers (515F: 5′‐ GTGCCAGCMGCCGCGGTAA‐3′ and 806R: 5′‐ GGACTACHVGGGTWTCTAAT‐3′). The thermal reaction conditions were 94°C denaturation for 5 min; 30 cycles of 94°C for 30 s, 54°C for 30 s, and 72°C for 30 s; and a final 10 min elongation step at 72°C. The PCR products was excised from a 1.2% agarose gel and purified using an AxyPrep DNA Gel Extraction Kit (AXYGEN Inc., Union City, CA, USA). Each purified PCR product was subjected to Illumina‐based high‐throughput sequencing (BGI, Shenzhen, China).

### 2.2 Bioinformatic analysis

To obtain more accurate and reliable results in the subsequent bioinformatic analysis (Fadrosh, Bing, Gajer, & Sengamalay, 2014), the raw data were preprocessed to get clean data using an in‐house procedure as follows: The sequences that contained more than two mismatches to the primers or more than one mismatch to the barcode were discarded, and reads of <50 bp were removed. If the window average quality value was <20, the end of the read sequence was truncated from the window, and the reads with a final read length <75% of the original read length were removed. The clean data were clustered with 97% similarity using USEARCH (v7.0.1090) (Edgar, 2013), and the operational taxonomic unit (OTU) sequences were searched against the RDP (ribosomal database) using RDP classifier (v2.2) (Cole, Wang, Fish, & Chai, 2014) with a confidence threshold of 60%. The alpha diversity index for each sample was determined using Chao1 (total species richness), ACE (abundance‐based coverage estimator) and the Shannon index in Mothur (v1.31.2) (Schloss, Westcott, Ryabin, & Hall, 2009). Based on the weighted UniFrac distance, to display the differences of OTU composition between the gut, sediment, and water, principal component analysis was used to construct a two‐dimensional (2‐D) graph to summarize the factors mainly responsible for this difference; similarity was high if two samples are closely located by package “ade4” of software R (v3.1.1). A heatmap was drawn based on the relative abundance of each species in each sample from the sediment, water, and gut samples. The Kruskal–Wallis test was used to analyze the bacterial composition and abundance (average abundance > 0.01%) in samples. The biological replicates were merged, and finally, the differential flora was visualized using the igraph package in R.

## 3 RESULTS

### 3.1 General analyses of high‐throughput sequencing

**Figure 1:**
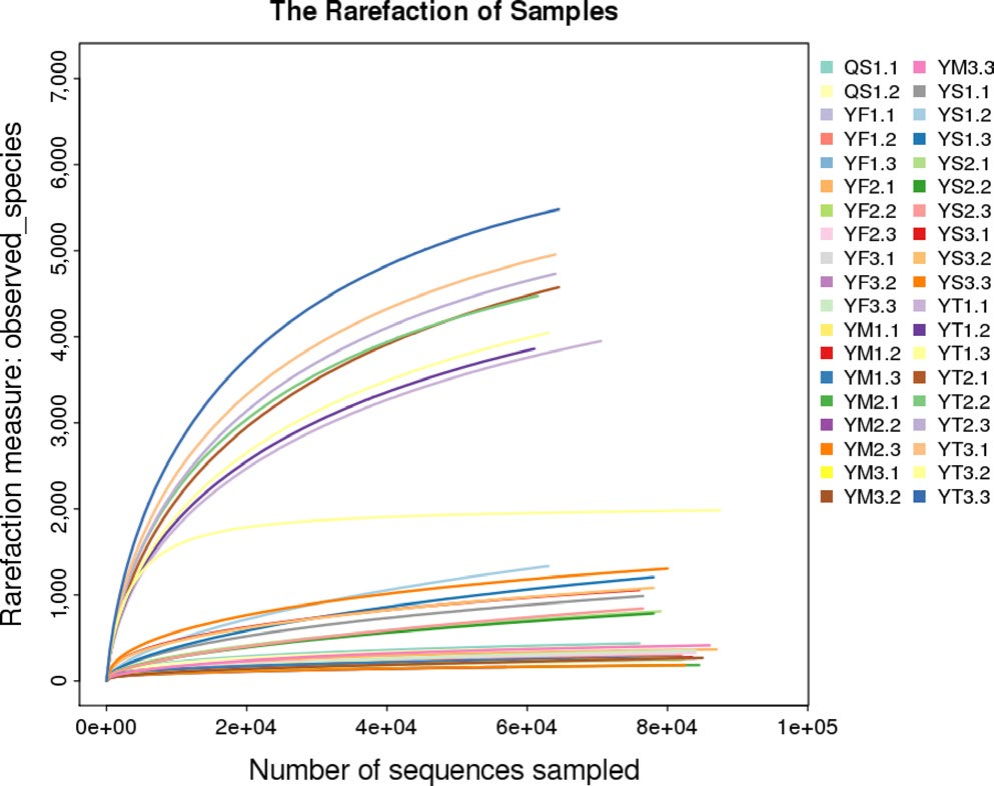
Rarefaction analysis of the samples

**Figure 2:**
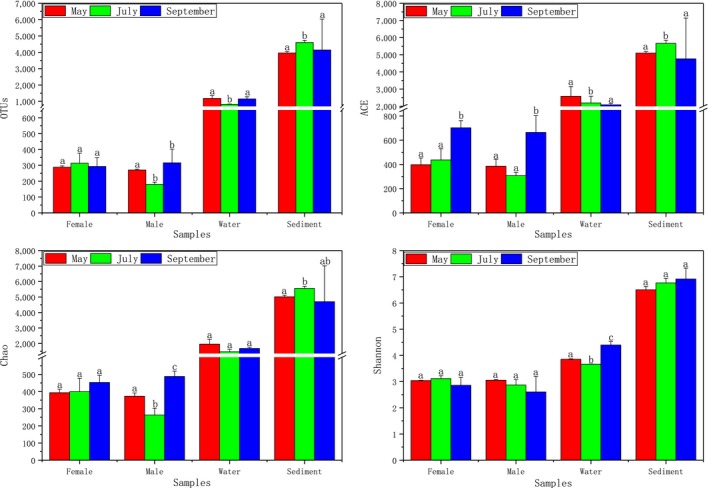
Comparison and analysis of bacterial composition richness (the OTUs, Chao index, and ACE index) and diversity (the Shannon index) in the samples. The alpha diversity values were compared in the female group, the male group, the water group, and the sediment group. OTU, Operational taxonomic unit; ACE, abundance‐based coverage estimator

After removing the low‐quality reads, a total of 2,916,923 valid reads and 9,814 OTUs were obtained from 38 samples. Among them, 57,425–87,343 were collected for intestinal bacterial community analysis, and 61,245–87,602 were collected for environment‐associated bacterial community analysis. The rarefaction curves showed that sufficient sampling depth was achieved for each sample (Figure 1). The alpha diversity score was applied to analyze the complexity of species diversity for the water, sediment, and guts of crabs (Figure 2). OTU numbers, ACE, Chao, and Shannon index can reflect the species richness of a community, and the rarefaction curve based on the three values could also be used to evaluate whether the produced data are sufficient to cover all species in the community (Schloss et al., 2009). In addition, the Shannon index can reflect the species diversity of the community. As shown in Figure 2, the bacterial community diversity in the environmental samples was higher than that in the gut samples. Moreover, the bacterial community diversity was higher in female crabs than in the male crabs. During the breeding cycle, the bacterial diversity showed a decreasing trend, with the bacterial community becoming more stable as the crab aged.

### 3.2 Microbial community compositions and dominant species

**Figure 3:**
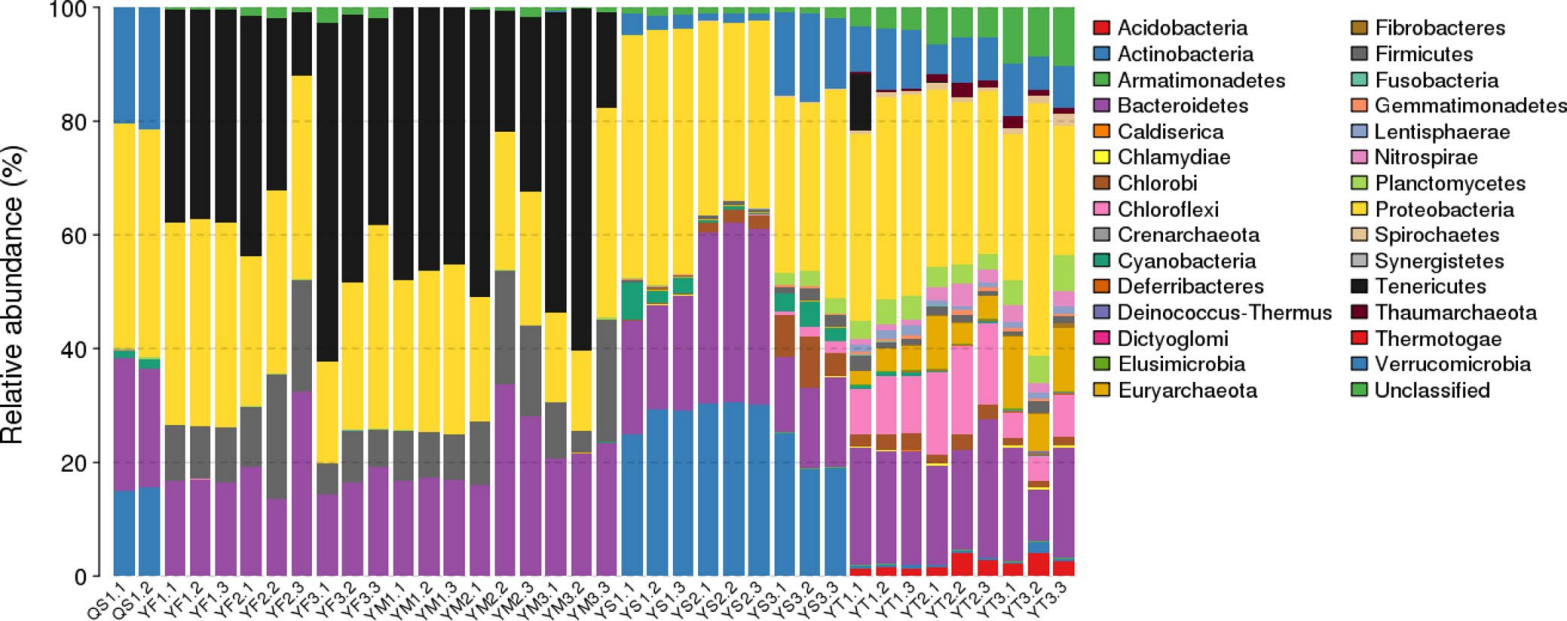
Frequency distribution of bacterial phyla in water, sediment, and intestines. QS represents the original water samples. YF1, YF2, and YF3 represent juvenile, young, and adult, respectively, in the female group. YM1, YM2, and YM3 represent juvenile, young, and adult, respectively, in the male group. YS1, YS2, and YS3 represent May, July, and September samples, respectively, in the water group. YT1, YT2, and YT3 represent May, July, and September samples, respectively, in sediment group

The phylogenetic classification of sequences from all samples identified 30 different phyla or groups (Figure 3). In the sediment samples, the bacteria were dominated by Acidobacteria (1.23%–4.1%), Bacteroidetes (9.07%–24.26%), Chloroflexi (4.34%–15.51%), Nitrospirae (0.88%–4.07%), Planctomycetes (2.81%–6.4%), Proteobacteria (22.6%–44.41%), and Verrucomicrobia (5.38%–10.76%). These seven phyla accounted for 68.6%–83.42% of the total reads. The water samples were dominated by the phyla Acidobacteria (18.63%–30.49%), Bacteroidetes (13.36%–31.57%), Chlorobi (0.17%–8.86%), Cyanobacteria (0.38%–6.47%), Proteobacteria (29.44%–44.95%), and Verrucomicrobia (1.27%‐15.45%), which represented 90%–98.1% of the total reads. The intestinal dominant bacteria included four phyla, including Bacteroidetes (13.4%–33.8%), Firmicutes (3.84%–21.97%), Proteobacteria (14.06%–36.83%), and Tenericutes (11.06%–60.14%). These four phyla represented 97.14%–99.87% of the total reads.

Female and male crabs had the same dominant bacterium but differed in the proportion of bacteria at the three time points. It is worth mentioning that the Proteobacteria and the Tenericutes account for 59.33%–74.18% of the bacteria in the female crabs and 57.37%–74% of those in the male crabs. Among them, the minimum values of the two phyla appeared in the youth stage, and the differences between May and September were not significant. Proteobacteria in female and male crabs were significantly different between the juvenile and youth stages (*p *<* *0.05), but there was no significant difference in the adult stage. For the Tenericutes, there was a significant difference between female and male crabs in the juvenile stage only.

### 3.3 The gut microbial composition in crab

**Figure 4:**
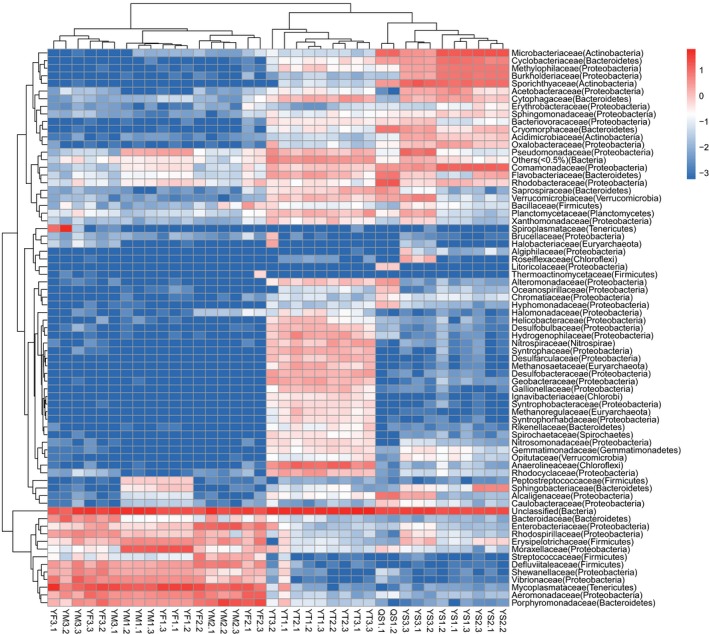
Heatmap analysis of the bacterial species in all samples. The color of the bar represents the abundance of each bacteria species in all samples. The longitudinal clustering indicates the similarity of all species among different samples

To determine the changes in the intestinal microbiota in response to the culture environment during the breeding period, we performed hierarchical clustering using the abundance of each species at the family level. The results showed that the gut microbiota, sediment, and water environment samples have different bacteria and different abundances (Figure 4). In the intestine of crabs, four bacteria, *Aeromonas* (Proteobacteria), Defluviitaleaceae (Firmicutes), *Candidatus Bacilloplasma* (Tenericutes), and *Dysgonomonas* (Bacteroidetes) are the four dominant bacteria in the intestine during the entire breeding cycle. This result is consistent with the dominance of the bacterial flora. Notably, the bacteria Moraxellaceae (Proteobacteria) and Pseudomonadaceae (Proteobacteria) were endemic in the juvenile stage. Similarly, Enterobacteriaceae (Proteobacteria) and Streptococcaceae (Firmicutes) were unique to the youth stage, and Enterobacteriaceae (Proteobacteria) had low abundance in female crabs. Bacteroidetes (Bacteroidetes) and Rhodospirillaceae (Proteobacteria) were specific to the adult stage, and among them, Rhodospirillaceae (Proteobacteria) showed low abundance in female crabs.

The four dominant bacteria showed significant changes in abundance during the life cycle of the crabs, with a similar abundance in juvenile and adult stage, but a higher abundance in the youth stage. Although male and female crabs showed consistent trends, the four main bacteria were consistently more abundant in the female crabs than in the male crabs.

### 3.4 Unique and shared bacteria groups between gut microbiota and the environment

The Kruskal–Wallis differential test was performed on the data, and biological abundance was merged by extracting the abundance of different species. Compared with the original water, the numbers of differentially present in May, July, and September in the gut microbiota were 26, 23, and 0, respectively. Compared with the corresponding environmental bacteria in the breeding pond, the numbers of differentially present bacteria in the gut microbiota were 103, 107, and 68.

**Figure 5:**
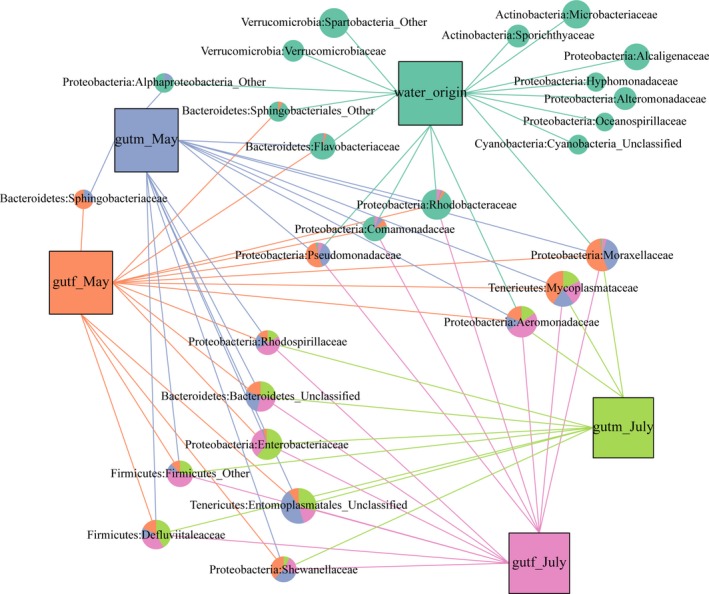
Network analysis visualizing the unique and shared bacterial groups between the intestinal microbes and in the original water microorganisms. The node size represents the abundance, and the data for common bacteria are drawn as pie charts according to their different proportions. If the abundance of the bacteria in the sample was <0.1%, only lines are used

**Figure 6:**
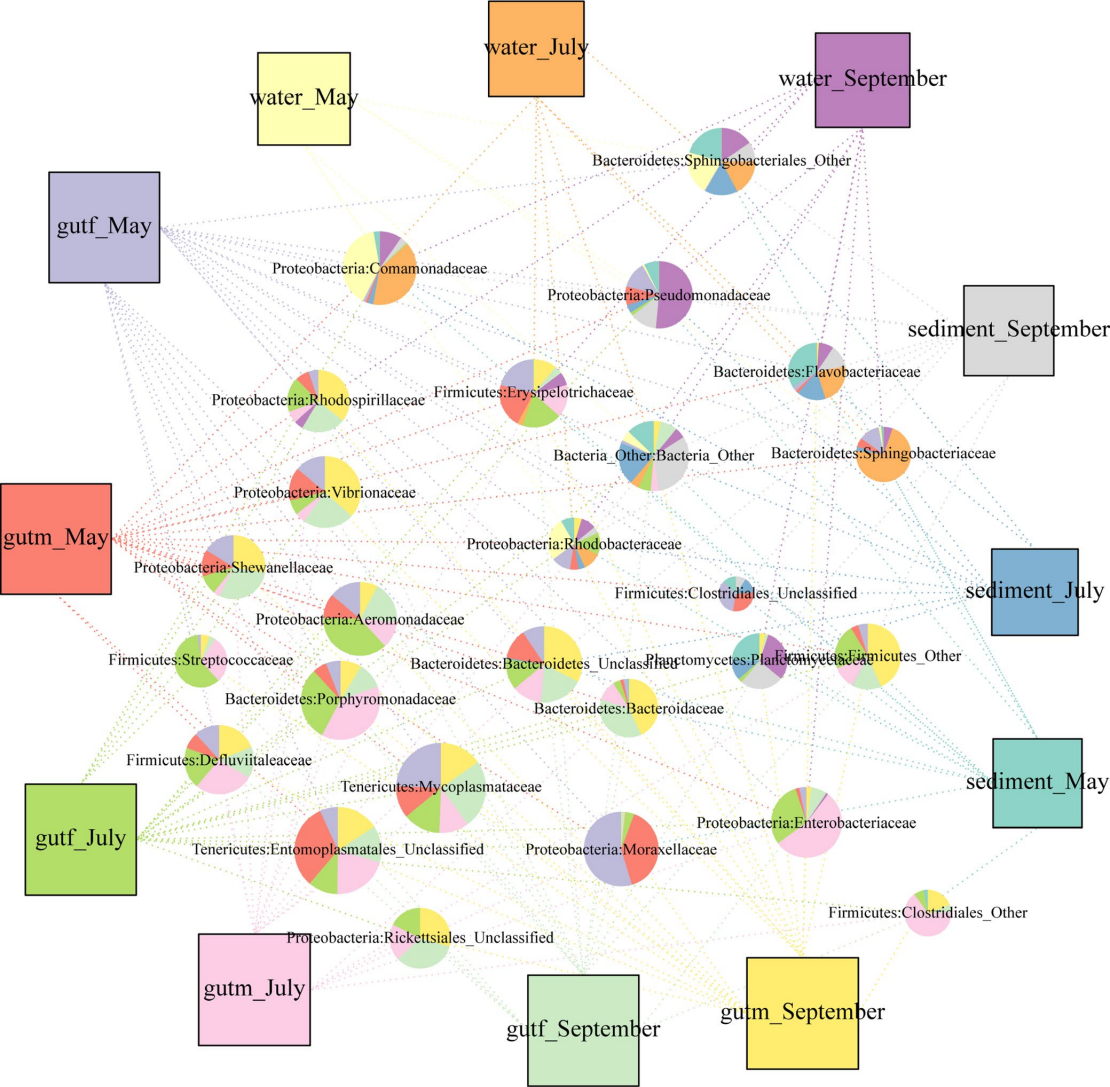
Network analysis visualizing the shared bacterial groups between the intestinal microbes and the environment microorganisms. The node size represents the abundance, and the data for the common bacteria are drawn as pie charts according to their different proportions. If the abundance of the bacteria in the sample is <0.1%, only lines are used

To understand the influence of the environment microbiology on the intestinal microbiology, the bacteria in the origin water sample and the corresponding environmental samples were analyzed for the difference in abundance compared with those in the intestinal microbiota of crab, and the results were visualized using a network association diagram (Figures 5 and 6). As shown in Figure 5, the number of bacteria common to the intestine and origin water sources was eight in May; however, in July, the number was five, while in September there were no bacteria common to the intestine and the original water sources. Interestingly, the common bacteria in May belonged to the phyla Verrucomicrobia, Proteobacteria, and Bacteroidetes; however, in July, the common bacteria belonged only to the Proteobacteria. Among the eight bacteria common to the environment and the intestinal tract, three of them were Aeromonadaceae (Proteobacteria), Pseudomonadaceae (Proteobacteria), and Moraxellaceae (Proteobacteria). The original water had a total of nine unique bacteria, belonging to Verrucomicrobia, Actinobacteria, Proteobacteria, and Cyanobacteria.

Figure 6 shows the unique and shared bacteria between the intestinal microbial bacteria of crab and the corresponding environment. In the sediment, microbes associated with intestinal bacteria decreased from 14 in May to 10 in September. The intestinal abundance of these bacteria also declined as the culture time increased. Compared with the sediment microorganisms, the intestinal microflora increased from 11 in May to 15 in September, and four of the resident bacteria also appeared to have no influence on the microbes in the sediment environment. In the water environment, the number of bacteria related to intestinal microbes increased from 6 in May to 11 in September, at which point the microbial communities basically overlapped with the sediment microorganisms. Similarly, the resident bacteria of the intestinal tract still have no effect on the environmental microorganisms.

## 4 DISCUSSION

Aquatic animals have greater differences in their life history and living environment than terrestrial animals (Wong & Rawls, 2012). Their intestinal microflora structure is more diverse and complex than terrestrial animals. For aquatic organisms, the factors that can affect the structure of the intestinal flora are the genetic background, physiological state, and biological habits (Ni, Yu, Zhang, & Gao, 2012; Xiong, Dai, Zhu, & Liu, 2016; Xiong, Wang, Wu, & Qiuqian, 2015). The physiological state can include the health status of the aquatic organisms, their feeding status, and age. Studies on the health status of aquatic organisms have commonly detected differences in sex and body weight (Li, Yan, Einar, & Wu, 2016) and in feeds (Akter, Sutriana, Talpur, & Hashim, 2016; Giannenas, Karamaligas, Margaroni, & Pappas, 2015; Hao, Wu, Jakovlić, & Zou, 2017) and feeding methods (Rungrassamee, Klanchui, Maibunkaew, & Chaiyapechara, 2014; Tzeng, Pao, Chen, & Weng, 2015; Wang, Zhang, Li, & Lin, 2016). The differences in the gut microbiota at different ages and the exploration of the core bacteria have been studied frequently for human and model organism intestinal microbes; however, relatively little is known regarding gut bacteria in aquatic organisms. Filling this knowledge gap is important to understand the relationship between aquatic organisms and intestinal microbes and to develop targeted probiotics.

The development of the relationship between intestinal microorganisms and the intestines is poorly understood (Fraune & Bosch, 2010). The intestines are important organs of aquatic organisms, and their growth and development are similar to other organs. For aquatic organisms, determining the exact composition of the gut microbiota is the first step in exploring the function of the bacteria and provides reliable basic data to reveal the relationship between the bacteria and the host's physiological metabolism. During the breeding process, aquatic organisms experience an important physiological transformation from the juvenile to the adult. However, the exploration of microbial composition at different ages has been performed more frequently in model organisms (Stephens, Burns, Stagaman, & Wong, 2016). Research on the intestinal microbes of crabs has concentrated on the exploration of the community structure and dominant bacteria in the adult stage, while research is lacking on the structure of intestinal microflora in other life stages.

From May to September, the three typical stages of the breeding period were selected to represent the juvenile, the young, and the adult *E. sinensis* and to explore the differences and similarities of their intestinal microbes. The results showed that the dominant bacteria in the three stages of the crabs were Bacteroidetes, Firmicutes, Proteobacteria, and Tenericutes. These four bacteria phyla represent a great proportion of the intestinal microbes; the female crab in the lowest proportion was 99.68%, and the lowest proportion was 99.58% in male crabs. Among them, Proteobacteria and Tenericutes occupy a large proportion of the dominant phyla. The ratios in the juveniles, young adults, and adults of the two phylum females were 73.21%, 59.33%, and 74.18%, respectively, while the proportions in male crabs were 74.64%, 57.38%, and 65.46%. This is similar to the results of previous studies. However, in a study of the growth of the microbial community of white prawn (*Litopenaeus vannamei*), which is also used as model crustacean, its dominant phyla were Proteobacteria, Bacteroidetes and Actinobacteria, of which Proteobacteria and Bacteroidetes are the two dominant phyla (Huang, Li, Wang, & Shao, 2016). For zebrafish intestinal bacteria, which is often used as a model animal, the dominant phyla were Proteobacteria and Tenericutes, with the Proteobacteria occupying a great proportion during zebrafish development (Stephens et al., 2016). Compared with those in higher animals, the dominant intestinal bacteria of aquatic organism are the Fusobacteria and Proteobacteria, whereas Bacteroidetes are relatively rare (Kostic, Howitt, & Garrett, 2013). However, in the Chinese mitten crab, the dominant phyla are Proteobacteria and Tenericutes. Thus, for the intestinal microbes, their dominant phyla are distinguished from those of crustaceans, which are closely genetically related, but similar to those of zebrafish, which is a vertebrate. Intestinal bacteria in zebrafish are related to host nutrition absorption, and research into their inherent immunity is relatively advanced, which has provided valuable experience for exploring the relationship between the host intestinal bacteria and nutrient absorption, immune response, and other aspects in crabs. The crabs used in the experiment were fed with the same feed, which eliminated interference from different foods.

Interestingly, for both male and female crabs, the proportion of Proteobacteria gradually decreased with age and the proportion of the Tenericutes showed a trend of decreasing first and then increasing. Comparing the intestinal microflora of the male and female crabs, the results showed that numbers of Proteobacteria were significantly different from the juvenile and to the youth stage, while Tenericutes was only significantly different in the juvenile stage, and there was no significant difference between the juveniles and adults. The commercial value of male and female crabs and the individual product specifications display huge differences in the commercial benefits. It would be interesting to explore the gender differences in the influence of these two phyla on the management of the production and breeding of males and females. Bacteroidetes and Firmicutes had the highest proportion in the youth stage, but maintained a lower proportion in both juveniles and adults; however, there was no significant difference in their proportions between the sexes. The association and regulation of the dominant bacteria in the host crab require further research to analyze their status and function among the intestinal microbes of the *E. sinensis*.

Comparing the microbial diversity of intestines at three ages, we found that the diversity of the gut microbiota was the highest in juvenile crabs and that the diversity decreased with age, which was similar to the results in zebrafish and African turquoise killifish (*Nothobranchius Furzeri*), which maintain high diversity in the juvenile gut (Smith, Willemsen, Popkes, & Metge, 2017; Stephens et al., 2016). The relationship between the diversity of intestine microbes and the individual growth of animals is not clear. Some scholars hold the view that an increase in the diversity of the flora would promote the growth of animals, whereas other scholars believe that the growth of animals would be limited. Therefore, further studies of the influence of microbial diversity on host growth and development and the development of specific bacterial populations are required.

The long‐term and stable colony of the “indigenous” bacteria in the host intestines has important implications for the physiological metabolism, nutrient uptake, and the immune response. In this study, four bacterial groups were identified during the three age stages of the *E. sinensis*:* Aeromonas* (Proteobacteria), Defluviitaleaceae (Firmicutes), *Candidatus Bacilloplasma* (Tenericutes), and *Dysgonomonas* (Bacteroidetes), three of which were identified as genera, and one was identified as a family. The more interesting phenomenon is that these four bacterial colonies belong to four dominant bacteria phyla. For the only species identified as Family, the Defluviitaleaceae, which belongs to the Firmicutes, has not been found in the intestinal tract of aquatic organisms and was found in the human gastrointestinal tract disease (Dong, Wang, Liu, & Yang, 2017). In this study, the population abundance of Defluviitaleaceae in young crabs was higher than that in juvenile and adult crabs, while the growth curve of these bacteria in the young population showed an exponential increase. Therefore, it remains to be further studied whether this bacteria group plays a role in the intestinal tract of the crabs to maintain physiological functions for gastrointestinal stability and nutrient absorption.

Two species, *Candidatus Bacilloplasma* (Tenericutes) and *Dysgonomonas* (Bacteroidetes), were the dominant of intestinal bacteria in this study and previous study, at the same time, it is worth mentioning that *Candidatus Bacilloplasma* is the “indigenous” populations of the intestinal, which is also consistent with previous studies on the intestinal bacteria of crustaceans (Kostanjsek, Strus, & Avgustin, 2007). In the study of the gut microbiota of shrimp containing pathogenic bacteria, researchers found that the intestinal abundance of *Candidatus Bacilloplasma* increased significantly in pathogenic shrimp. In the present study, *Candidatus Bacilloplasma* had the lowest abundance at the youth stage in *E. sinensis*, but it maintained a high abundance in the juvenile and adult stage. The youth stage is the fastest growing stage and the most advanced stage in terms of the immune system in the breeding cycle, so whether *Candidatus Bacilloplasma* is a potential pathogen in the intestine of the crabs, and its status and function among the intestinal bacteria remain to be further explored. Bacteroidetes has a corresponding abundance in the intestines of fish and shrimp (Baldo, Riera, Toomingklunderud, & Albà, 2015; Rungrassamee et al., 2014; Zhang, Sun, Chen, & Yu, 2014); however, its function has not been determined. The genus *Dysgonomonas* has a certain abundance in the gut of cockroaches and termites (Mikaelyan, Thompson, Hofer, & Brune, 2015). It is a common genus of termites intestines and plays an important role in assisting termites to digest lignocellulose, in immunity, and in reproduction (Brune, 2014; Fraune & Bosch, 2010; Scharf, 2015; Su, Yang, Huang, & Su, 2016; Warnecke, Luginbühl, Ivanova, & Ghassemian, 2007; Werren, Baldo, & Clark, 2008). *E. sinensis* belongs to the crustacean class and the arthropod phylum, as do termites; and in crabs, the *Dysgonomonas* genus reached its highest abundance in the youth stage and was low in the juvenile and adult stages. During the sampling, the samples of the juvenile and young populations showed significant differences in their specifications; therefore, whether the function of the bacteria is related to the intestinal nutrient absorption and the level of immunity, and whether they are beneficial bacteria, requires further exploration.

Compared with previous studies, a more special kind of *Marinifilum* bacterium appeared in the gut microbiota of *E. sinensis*, with high abundance in juvenile stage, but lower abundance in the young and adult stages. The samples of this study originated from Qinghai Province, which has inland lakes with high salinity and pH, and the low temperatures. However, the intestinal microflora of wild crabs collected at the same sampling time did not contain this bacterium (data not published). Previous studies have found that crabs sampled on Chongming Island have this bacterium, but they were not found in samples from Taihu Lake (Chen et al., 2015; Zhang et al., 2016). Comparing the changes in the living environment and the factors influencing the different environments, it was noted that the presence of *Marinifilum* bacterium is the result of the adaptation of crabs to high salinity, being a special bacterial flora adapted to high pH environments. The status and function of this group of bacteria in the physiological metabolism of crabs requires further exploration.

The aquatic environment has long‐term effects on aquatic organisms. The differences in intestinal bacteria between different aquatic organisms have been studied in the black tiger shrimp (*Penaeus monodon*) and the White Prawn (*L. vannamei*) (Chaiyapechara et al., 2012; Zhang et al., 2014). The comparisons between the intestinal bacteria of crustaceans in different culture environments revealed obvious differences. However, there have been few studies of the correlation between the environment and aquatic intestinal bacteria, especially studies of influence of the environment on the intestinal flora during the breeding cycle. In this study, the correlation between the original water bacteria group and the intestinal bacteria and the correlation between the bacteria group of the cultured pond and the intestinal bacteria were analyzed and visualized. The results showed that with increasing time after leaving the source environment, the number of water‐related intestinal bacteria gradually decreased. In the ponds, with increasing age and crab breeding time, environment‐related intestinal bacteria also gradually declined. To the best our knowledge, this is first study to perform environmental bacteria correlation analysis and crab intestinal bacteria analysis at the breeding stage. The results showed that with increasing cultivation time, the influence of the external environment on the intestinal microorganisms of these aquatic organisms gradually reduced. In addition, the proportion of the core bacteria seemed to depend on the physiological stage, not the external environment. Recent studies have shown that the correlation between the host intestinal microflora and the external environment may change in response to variations in the host's physiology and the external environment (Alberdi, Aizpurua, Bohmann, & Zepeda‐Mendoza, 2016), which corroborates the results of this study.

In summary, the present study investigated the development of the intestinal flora of *E. sinensis* in three growth stages and identified the core bacteria from the juvenile to the adult stage: *Aeromonas* (Proteobacteria), Defluviitaleaceae (Firmicutes), *Candidatus_Bacilloplasma* (Tenericutes), and *Dysgonomonas* (Bacteroidetes). The classification level is accurate to the family and genus levels. This result will not only help researchers understand the assembly of the intestinal microflora of *E. sinensis*, but also provides reliable theoretical support for follow‐up studies of host–microbiota relationships and the development of probiotics. It will also help to improve the precise management of crabs in aquaculture. At the same time, through the study of the correlations between the bacterial populations of the source water, the environment bacteria in the pond, and the intestinal microbiota of the crab, we showed that the adaptability of the crab to the external environment increased with increasing culture time and that the core bacteria were not affected by the external environment. Identifying the core group of crab bacteria provides the basis to study the relationship between the specific bacteria and the physiological metabolism of *E. sinensis*. Furthermore, the impact of food sources on the structure of crab microbial populations requires further research.

## CONFLICT OF INTEREST

The authors declare that they have no conflict of interests.

## AUTHORS CONTRIBUTION

CHW, YFZ, DWL, YG, YY conceived and designed the experiments. CHW, YFZ, DWL, YG. performed the experiments. CHW, YFZ, HL, YY. analyzed the data. YFZ, DWL, YY. contributed reagents/materials/analysis tools. CHW, YFZ, YY wrote the paper.

## ETHICAL STATEMENT

Animal use was approved by, and was under the supervision of, the Freshwater Fisheries Research Center, Chinese Academy of Fishery Sciences (FFRC, CAFS).

## Data Availability

In the article, HiSeq Illumina sequencing raw sequence reads data: https://www.ncbi.nlm.nih.gov/bioproject/PRJNA451256, https://www.ncbi.nlm.nih.gov/bioproject/PRJNA451312.
